# The role of threats in animal cooperation

**DOI:** 10.1098/rspb.2010.1241

**Published:** 2010-08-26

**Authors:** Michael A. Cant

**Affiliations:** Centre for Ecology and Conservation, University of Exeter, Cornwall Campus, Tremough, Penryn, Cornwall TR10 9EZ, UK

**Keywords:** negotiation, within-group conflict, reproductive skew, mutualism, biparental care, cooperative breeding

## Abstract

In human societies, social behaviour is strongly influenced by threats of punishment, even though the threats themselves rarely need to be exercised. Recent experimental evidence suggests that similar hidden threats can promote cooperation and limit within-group selfishness in some animal systems. In other animals, however, threats appear to be ineffective. Here I review theoretical and empirical studies that help to understand the evolutionary causes of these contrasting patterns, and identify three factors—impact, accuracy and perception—that together determine the effectiveness of threats to induce cooperation.

## 1. Introduction

An alien observer of human behaviour might take some time to realize that our social interactions are shaped to a large extent by hidden threats. Threats of fines and incarceration serve to maintain law and order, while threats of social punishment such as ostracism or peer criticism encourage us to conform to social norms [1–3]. But an observer would find it difficult to identify these threats because punishments are triggered only when the social rules they enforce are broken. The most effective threats are those that rarely need to be carried out, and so are least likely to be noticed by an observer.

Recent theory and experimental studies suggest that hidden threats may play a similarly important role in shaping the social behaviour of animals. In particular, threats to terminate a potentially profitable interaction may limit the level of selfishness in cooperative groups [4–6]. Nature abounds with examples of cooperation where animals can interact to produce a mutual inclusive fitness benefit: examples include interspecific mutualisms [7,8], biparental care systems [9,10], cooperatively breeding species [11–13] and parents with their offspring [14]. However, conflict arises in these interactions because each individual is selected to maximize its share of the inclusive fitness profits of cooperation at the expense of its social partners. For cooperative associations to form and remain stable, participating individuals must resolve this conflict in such a way that each prefers to continue the interaction rather than pursue alternative *outside options*, such as leaving, evicting or eliminating their partner [5,15,16]. Outside options place a limit on the level of exploitation that an individual will tolerate before it does better to break up the interaction. Consequently, threats to terminate a cooperative interaction (‘exit threats’) can, if they are effective, curtail the level of within-group selfishness and prevent the dissolution of potentially profitable associations.

Here I describe theory that helps to understand how threats can constrain selfishness in biological interactions, and review studies that illustrate the success and failure of threats to induce cooperation in a variety of systems. These examples reveal that, in many animals, observed acts of punishment and control may represent just the tip of the iceberg of forces shaping social structure and behaviour.

## 2. Threats and social control: theory

**Figure 1.**
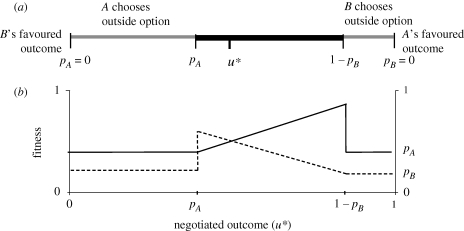
(*a*) Fitness surplus and bargaining set in two-player cooperative association. Both parties benefit from cooperation if they can negotiate a settlement u*, which lies in the bargaining set (the thick black line). In the case shown, the two parties are of equal ‘bargaining power’, so u* is located at *p*_*A*_ = *p*_*B*_ = 0.5. (*b*) Fitness payoffs accruing to player *A* (solid line) and player *B* (dotted line) as a function of the negotiated outcome u*.

To focus our discussion, consider the interaction between two individuals, *A* and *B*, who can interact in some way that produces a net direct fitness profit. The two individuals face conflict because each is selected to increase its share of the total profits (or fitness ‘surplus’) at the expense of its partner's share. This surplus is represented in figure 1*a* by a line with player *A*'s preferred outcome at one end and player *B*'s preferred outcome at the other. Each player's fitness payoff (or inclusive fitness payoff in the case of relatives) resulting from the interaction is assumed to be an increasing function of their share of the total profits. The figure shows the simplest case, where fitness is directly proportional to this share, the fitness surplus is constant irrespective of how it is distributed and the players are unrelated (so that indirect fitness effects can be ignored).

Layered on top of this we need to consider the alternative outside options that may be available to the two individuals. Each of the two players may have the option to terminate the interaction to pursue these outside options (for example, by leaving, evicting or eliminating their partner), in which case they can expect payoff *p*_*A*_ and *p*_*B*_, respectively. If a player has more than one outside option, we need only plot the highest-paying outside option on our line, since other outside options will never be chosen. If none of a player's outside options are more profitable than remaining with zero share of the fitness profits, then that player effectively has no outside option. The zone between the outside options of the two players represents the set of outcomes, where both players profit from the interaction. In economic models this is called the ‘bargaining set’ [17]. To avoid unnecessary duplication of terms I follow this usage here.

How will the two individuals resolve conflict over the division of the ‘pie’ of profits? Theoretically, we can distinguish two distinct ways by which animals might influence this competition: (i) bargaining and (ii) the use of threats. The distinction lies in the assumed game structure of the interaction between players. A *bargaining* process involves a potentially infinite exchange of actions or signals that may converge to some equilibrium or behavioural ‘settlement’. By contrast, *threats* arise when one player can make a ‘last move’ to terminate the interaction or inflict a lasting cost on the other player. The threat to exercise this last move generates an incentive for the other player to exercise restraint in its claims on the fitness surplus. Both these processes are elements of behavioural ‘negotiation’ and presuppose the ability of each individual to respond on a behavioural timescale to changes in the behaviour of the other (i.e. they invoke evolutionary ‘rules for responding’ rather than fixed genetic strategies; see [10,18]).

The process of bargaining might take various forms in nature. In cooperative breeders, for example, dominant and subordinate breeders might engage in aggressive interactions to claim or defend resources required for reproduction, or to signal a willingness to escalate in conflict [19–21]. Offspring may engage in costly acts or signals to claim additional resources from parents or siblings [22,23]. In species that exhibit biparental care, a male and female parent may bargain by mutual adjustment of provisioning effort [10,18]. The ability of one party to shift the resolution in its own favour (which we can call its ‘bargaining power’) will depend on asymmetries in quality or the ability to sustain costs during bargaining [10,24]. There may also be asymmetries in bargaining power that have nothing to do with their individual attributes; for example, it may be cheaper for one party to increase its level of service or resource production than the other [4]. In cooperative species it may be easier for a subordinate helper to shirk than it is for a dominant to force it to help. This latter example illustrates the point that, depending on the context, one party may be a stronger bargainer even though it is physically weaker and socially subordinate to the other [3].

What if the bargained resolution lies outside the bargaining set? In this case one of the players possesses a credible threat to break up the association. The threat is credible because it is in the threatener's own interest to exercise its outside option when its expected payoff from bargaining is less than its outside option. This threat can affect the resolution by forcing the other player to ‘ease off’ in bargaining until the threat is no longer credible; that is, to concede just enough fitness to the other player to match its relevant outside option. If, on the other hand, the bargained resolution lies within the bargaining set, neither player's threat to exercise their outside option is credible because it would not pay either of them to carry out their threat. In economics this is sometimes referred to as *the outside option principle*: outside options are relevant only where they yield a higher payoff than can be obtained through bargaining [25]. A corollary of the argument is that only one player's outside option can be relevant at a time, because if both players receive less at the negotiated equilibrium than they would gain from pursuing their outside option, there is nothing to be gained from trying to cooperate in the first place. The outside option principle can be used to develop ‘synthetic’ models of conflict resolution that incorporate threats and bargaining in the same framework [5].

We expect strong selection to avoid triggering threats because a stronger bargainer experiences a sudden drop in fitness if a threat is triggered. Figure 1*b* shows how the fitness of our two players changes across the threat threshold. In this example, assume that player *A* has greater bargaining power and can push the bargaining settlement across the threat threshold of player *B*. At this point, *B* decides to take up its outside option (say, to leave the group) and receives a payoff proportional to *p*_*B*_, but as a consequence of this decision player *A*'s fitness drops suddenly from 1 − *p*_*B*_ to its own outside option fitness *p*_*A*_. The threat threshold represents a fitness ‘cliff-edge’ for the recipient of the threat [26]. Player *A* does best to push player *B*'s share down as close to the threat threshold as possible, without triggering it. Both players can therefore gain from effective communication to avoid triggering the threat unnecessarily. In §3, I describe cases where there is good evidence of pre-emptive behaviour to avoid triggering a threat, indicating that credible means of communicating (or detecting) threats do exist in nature, at least in some systems.

## 3. Effective and ineffective threats in nature

The type of exit threats that are relevant in animal systems will depend on the availability of alternative partners or resources, and whether one party is able to control group membership or access to resources. I focus on three types of threat in particular: the threats to evict, depart or to attack the other player. In each case I review examples that illustrate the effectiveness (or lack thereof) of threats to influence behaviour.

### (a) The threat of eviction

Some of the clearest evidence of hidden threats at work comes from recent studies of fish size hierarchies [27–32]. In these species, group members exhibit a size-based queue in which the largest individuals are breeders and the rest are non-breeders, and there are often consistent size differences between individuals at adjacent rank [33]. Experiments have shown that these size differences between ranks are maintained because subordinates strategically adjust their growth rates to remain smaller than their immediate dominant [27,29,30]. The hypothesis is that subordinates adjust growth rates to avoid the threat of expulsion from the group [28]. However, evictions are rarely observed in nature, and there are other plausible explanations for size differences in a hierarchy; for example, it may be that low-rankers have less access to food for growth. How can we test whether the threat of eviction drives the formation of the size hierarchy?

**Figure 2.**
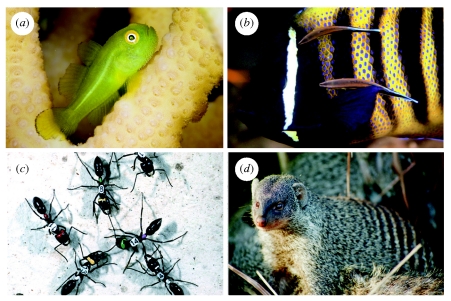
Effective and ineffective threats in nature. (*a*) The threat of eviction: in the coral-dwelling goby *Paragobiodon xanthosomus* subordinates adjust their growth to avoid triggering the threat of eviction by dominants [31,32]. (*b*) The threat of departure: studies of the cleaner fish *Labroides dimidiatus* and its clients suggest that the threat of departure can deter cleaners from ‘cheating’ (that is, feeding on client tissue rather than their ectoparasites [49–51]). (*c*) The threat of attack: subordinates of the queenless ant *Dinoponera quadriceps* appear to be deterred from challenging the dominant female by the threat of attack from nestmates. Dominant females mark challengers (such as female 14 in this photo) with a chemical that singles them out for ‘immobilization’ by other workers [66]. (*d*) An ineffective threat: in banded mongooses *Mungos mungo*, dominant females limit reproductive competition by evicting subordinate females from the group, but females do not exercise pre-emptive reproductive restraint to avoid eviction [39]. In this species, evicted females are often permitted to return, and even non-breeders are sometimes evicted, two factors that reduce the effectiveness of the threat of eviction as a deterrent. Photos: (*a*) Marion Wong; (*b*) Maxi Eckes; (*c*) Thibaud Monnin; (*d*) Roman Fuller.

The best way to test this idea is to ‘break the rules’ in order to trigger hidden threats. Just this type of experiment was performed by Wong *et al*. [31] working on the size hierarchies of the coral-dwelling goby *Paragobiodon xanthosomus* (figure 2*a*). To test whether observed size differences reflect the threat of eviction, Wong *et al*. paired individuals of different size in the laboratory and recorded their interactions. When there was a large size difference between the two individuals, the larger ‘dominant’ fish tolerated the presence of the smaller ‘subordinate’. However, when the difference in size between the two fish was smaller than the minimum difference observed in natural groups, dominants responded by forcibly evicting the subordinate. It was also clear why dominants stand to gain from evicting subordinates before they grow too large, since dominants paired with the largest subordinates often ended up being evicted themselves. In a second study using intact groups in aquaria, Wong *et al*. [32] showed that subordinates cease feeding as they approach the size threshold at which eviction is likely to be triggered. This indicates that these subordinates were able to detect the presence of the eviction threat and respond pre-emptively to avoid triggering it.

In these fish hierarchies, the threat of eviction is a powerful inducement to exercise restraint because subordinates place a high value on continued membership of the group, while dominants have little to lose by evicting them. The same factors apply in many cooperatively breeding vertebrates, particularly where the presence of subordinates threatens the reproductive monopoly of dominant breeders [34]. In meerkats, for example, subordinate females are forcibly evicted from the group by dominant females, and only allowed to return after the dominant has given birth [35,36]. Subordinate females that are pregnant at the time of eviction often abort their litter in the period when they are excluded from the group, lose weight and show signs of elevated endocrinological stress [37]. Consequently, eviction by the dominant female substantially reduces the probability that a subordinate will reproduce successfully. It is clear, however, that compared with fish size hierarchies, the threat of eviction is not a wholly effective deterrent in meerkats, since subordinates commonly reproduce, albeit at a much lower rate than dominants [38]. In banded mongooses, dominant females use eviction to limit the number of breeding females in the group, but there is no evidence that subordinates exercise pre-emptive restraint to avoid being evicted in the first place [39]. Threats may be less effective in social mongooses because eviction is often temporary and has less lethal consequences than it does in the social fish systems.

Finally, the threat of eviction plays a central role in ‘pay-to-stay’ models of helping behaviour. The idea here is that subordinates may be forced to pay ‘rent’ by helping in order to be tolerated in the group [40,41]. Again, the best evidence comes from a fish system, the cooperative cichlid *Neolamprologus pulcher*. In a field study helpers that were removed for periods of 4–6 hours were attacked or evicted upon their return, and those that were permitted to stay worked harder thereafter [42]. In the laboratory, breeders evicted helpers when they had little need for their help, and allowed them to return when help was required [43]; and helpers reduced their effort levels when they were provided with options to breed independently [44]. Finally, helpers that were experimentally prevented from defending the group against a predator (by denying them information of the predator's presence) responded by increasing their helping effort, which the authors argue may serve to appease the dominant and avert expulsion [45]. Note, however, that this manipulation to break the rules did not trigger eviction, contrary to the prediction of pay-to-stay models, although this may be because of the short-term nature of the manipulation. In other species, with a few rare exceptions (e.g. superb fairy-wrens [46]), there is very little evidence in support of pay-to-stay models. This can be attributed in part to the scarcity of experimental studies to manipulate helper effort [47]. However, it may also reflect the inefficiency of eviction as a strategy to punish helpers, even lazy ones. Eviction will be much more cost-effective when used to punish reproductive rivals or competitors that actively inflict costs on the evictor.

### (b) Departure threats

Where alternative partners or resources are readily available and there is no territorial or positional advantage from staying put, it may be more profitable simply to leave rather than contest a resource or attempt an eviction. The threat of departure is highlighted in biological market theory, in which the ability to exercise ‘partner choice’ is a main promoter of cooperative behaviour [6,48]. In the cleaner fish *Laboroides dimidiatus*, field observations suggest that the threat of departure by clients may induce cleaners to cooperate rather than cheat (i.e. to feed on ectoparasites rather than on preferred client tissue; figure 2*b*) [49]. Laboratory experiments support this hypothesis: cleaners quickly learn to be more cooperative with artificial ‘clients’ (Plexiglas plates containing food) that depart in response to ‘cheating’ (feeding on a preferred food type) compared with clients that do not [50,51]. Partner choice is argued to be a key driver of cooperation in other interspecific mutualisms [8,52], and in intraspecific contexts such as mating markets and sexual selection [53,54], grooming behaviour [55,56] and helping effort in cooperative breeders [57]. However, these studies have not manipulated cooperation or outside options experimentally, so definitive evidence of effective departure threats in these contexts is lacking.

In the study of cooperative breeding, the threat of departure forms the basis of classic ‘concession’ models of reproductive skew, which seek to explain variation in reproductive partitioning within groups [58,59]. These models suggest that where dominants gain from retaining subordinates in the group, they may do best to yield a share of reproduction as an incentive to keep them in the group. Unlike the case for biological markets, however, there is little evidence that departure threats influence the resolution of within-group conflict in cooperative breeders. For many cooperative breeders, the option to remain in the group as a non-breeder is often preferable to departure because subordinates can expect to inherit breeding positions in future, and because there are often tight ecological constraints on dispersal and independent breeding [60]. As noted, far from offering a staying incentive to keep subordinates in the group, dominants in many species go to considerable lengths to evict them. To date, two studies have managed to manipulate the availability of outside options experimentally to look for an effect on skew (on a social bee [61] and a cichlid fish [62]). Neither study found an effect on the level of reproductive sharing. Moreover, experiments to reduce the share of paternity obtained by subordinate males in cooperative breeders have never led to the departure of these males, as would be expected if they were ceded paternity to keep them in the group [19,63]. Overall, there is little evidence that the threat of departure is effective in reproductive competition in either insect or vertebrate cooperative breeders, although more experiments are needed.

### (c) Threats of attack

The third major type of threat is that of physical attack. Unlike eviction and departure, physical attacks do not necessarily lead to the termination of the interaction, unless fighting is lethal. Low-level aggression and dominance interactions might therefore be viewed as part of a bargaining process, rather than as a ‘last move’. However, where attacks inflict death, permanent damage or otherwise produce a step change in the fitness of the victim (figure 1*b*), the decision to attack is equivalent to an outside option, and may deter selfishness or induce cooperation in much the same way as a threat to break up the group.

Threats of attack may be directed against cooperative partners themselves, or against their offspring. For example, many social Hymenoptera queens and workers use aggression or egg-eating to deter their nestmates from reproducing (or developing into reproductives), a behaviour known as ‘policing’ (reviewed by Ratnieks and co-workers [64,65]). Policing is usually inferred from observations of aggression or oophagy (see table S1 in [64]), but, as with human policing, insect policing operates most efficiently via the use of threats. In the queenless ant *Dinoponera quadriceps*, for example, dominant breeders can prevent high-ranking subordinates from challenging their position by daubing them with a pheromone, which marks them out for attack and ‘immobilization’ by other workers [66]. This threat dramatically increases the potential costs of challenging to subordinates, and helps to stabilize the hierarchy in natural colonies [67,68]. In other species, queen-removal experiments suggest that the threat of physical attack helps to deter subordinates from becoming reproductively active (for example in hymenopterans [69–72] and naked mole rats [73–75]). In general, the formation of a stable dominance hierarchy presupposes the presence of effective threats (of attack or eviction) to deter challenges from lower-ranked individuals. Hierarchies reduce the costs of conflict precisely because they are stabilized by threats that rarely need to be exercised.

Threats of attack against offspring can also deter reproduction, particularly in species where each offspring represents a relatively large parental investment. In marmosets and meerkats, for example, dominant females sometimes kill the offspring of subordinates that reproduce. These acts occur infrequently, however, because the threat of infanticide is usually sufficient to deter subordinates from attempting to reproduce in the first place [76,77]. When subordinate mammals do breed, it is usually the oldest or largest females that do so [38], perhaps because they can defend their offspring more effectively or possess a credible threat of retaliation of their own. *A priori*, we would expect threats of infanticide to be much less effective in insect societies, where eggs can be produced and replaced very cheaply. Indeed, workers lay eggs even in systems where almost all of them are destined to be policed [65,78]. Nevertheless, across nine monogynous wasp species (plus the honeybee *Apis mellifera*), the average level of worker reproduction declined with the efficiency of policing [78], which is consistent with the hypothesis that policing involves an element of deterrence (what Ratnieks & Wenseleers [65] term ‘preventive policing’).

## 4. When will threats be effective?

It is clear from this brief survey that, first, with a few notable exceptions, evidence for the presence of effective hidden threats is scarce; and, second, that the number of studies that are designed (or could be expected) to detect the influence of threats is small. Where experiments to manipulate cooperation or outside options have been carried out (e.g. in fish size hierarchies, cooperative cichlids, cleaner–client systems and social insects), threats are often found to influence cooperation. Hopefully, further experimental studies of cooperative systems are forthcoming. Pending these we can synthesize the existing information to identify factors that are likely to promote the effectiveness of threats in biological systems. Three factors will be particularly important: the impact of a threat, the accuracy with which it is targeted and its perception (that is, the level of information about the threat on both sides).

First, the impact of a threat is the inclusive fitness cost that the transgressor stands to suffer if the threat is triggered. Threats will have greatest impact where the fitness value of outside options is low and when triggering a threat represents an irreversible final move in the interaction. This helps to explain why eviction threats are so effective in inducing growth restraint in the fish size hierarchies, and why evictions are so rarely observed. The cost of being evicted is undoubtedly extremely high in these fish systems as adult fish are not observed to move between existing groups [28,79]. In addition, it is very hard for a subordinate fish to ‘un-grow’ once it has crossed the threshold for eviction, so it must approach the threat threshold very cautiously indeed. In banded mongooses (figure 2*d*) and meerkats, by contrast, evicted females can ‘take back their move’ by aborting their litter, in which case they are readily accepted back into the group [36,39]. Similarly, a wasp worker whose egg is policed can always produce another one, at little fitness cost. These low impact and repeated punishments blur our simple distinction between negotiation and threats, and will have little lasting deterrent effect.

**Figure 3.**
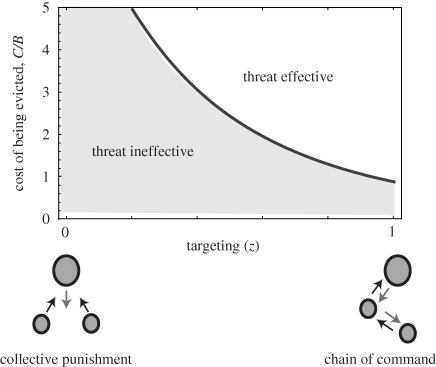
Effectiveness of threats to enforce cooperation in multi-member groups. The figure shows the results of a three-player model in which a single dominant can evict one of the two subordinates if either of them defects (redrawn from [39]). The figure shows the zones for which the threat is effective versus ineffective at inducing cooperation, as a function of accuracy with which it is targeted and the cost of being evicted. The cost *C* of being evicted is measured relative to the benefit *B* of defecting but escaping eviction. The targeting parameter *z* varies from 0 (implying that subordinates are equally likely to be evicted whether they choose to cooperate or defect) to 1 (implying that a subordinate who cooperates is never evicted). The case where *z* = 1 applies to groups that exhibit a hierarchical structure, such that each individual monitors and punishes its immediate subordinate (labelled ‘chain of command’ in the figure). As the accuracy of targeting declines, the threat of eviction rapidly becomes ineffective at enforcing cooperation. See [39] for details of the model.

Second, a threat needs to be accurately targeted so that transgressors suffer the consequences of their own actions. In the two-player ‘restraint’ skew model of Johnstone & Cant [34], the threat of eviction is effective at inducing reproductive restraint in a subordinate, because a subordinate that claims too large a share of reproduction is certain to suffer the cost of being evicted. In larger groups, by contrast, it may be difficult for a dominant to identify transgressors with certainty. Discrimination or targeting errors will weaken the effectiveness of a threat in two ways. First, transgressors may escape punishment. Second, targeting errors erode the incentive to cooperate because a non-transgressor may end up being punished anyway [80,81]. For these reasons, threats rapidly become ineffective as targeting accuracy declines ([39]; see figure 3). Threats will therefore be most effective in dyadic interactions, in groups in which there is a clear dominance hierarchy, or where there are other mechanisms by which cheaters can reliably be identified. In fish size hierarchies, for example, each subordinate is clearly identifiable to its immediate dominant at all times and can be singled out for eviction if it grows too large in size. In ants the threat of worker attack is effective as a reproductive deterrent because reproductively active workers can be detected from their signature cuticular hydrocarbons [71], or daubed with an identifying chemical mark [66].

Third, the effectiveness of a threat requires that both parties have information about the consequences of the threat and the conditions under which it would be carried out. Each party must have some estimate of the value of outside options relative to the payoff of continued cooperation in order to assess whether threats are credible and respond appropriately. In situations where individuals interact repeatedly or with a series of partners, this information could be gained through trial-and-error learning [50,82]. Alternatively, cooperators could signal to each other their willingness to exercise an outside option. In the example shown in figure 1*b*, player *A* has a strong incentive to heed any signals from *B* that it is about to exercise its threat, but at the same time *B* has an incentive to exaggerate its willingness to exercise a threat. Signals aimed at conveying an imminent threat will therefore lack credibility unless they are costly in some way to the signaller [3,83,84]. Facial signals, dominance displays and low-level aggression may credibly signal an impending threat of eviction or attack if they involve real costs to signallers. On the other hand, receivers may gain a strategic advantage if they can remain credibly ignorant of a threat signal or of the value of outside options. In *Polistes* wasps, for example, dominant foundresses rarely leave the nest, so they may remain insensitive to threats by their subordinates that are based on the value of outside options [60]. The topic of how and when threats are signalled, concealed or credibly ignored presents rich possibilities for future research.

I began by drawing an analogy between human and animal threats, but it is important to distinguish between the types of threat that are employed in human affairs and those we should expect to see in animals. In interactions among humans, one party can gain advantage by making a ‘strategic threat’—that is, by committing himself or herself to carry out a threat in the event of a transgression, even though there would no longer be an incentive to do so in the event that the transgression occurred [3,17,84]. For example, a kidnapper might threaten to kill a hostage unless a ransom is paid. However, for this threat to be credible, the kidnapper must establish a commitment to follow through with the execution in the event of non-payment, even though there would be little or no incentive to do so at that point (assuming, that is, no other cost of hostage release). In animals, it may be difficult for individuals to bind themselves to carry out threats which would yield no immediate gain (although McNamara & Houston [85] suggest some scenarios where this may be possible). If an animal carries out a threat, it is likely that there is an immediate or future benefit from doing so at that point; for example, because exercising the threat protects them from further exploitation. Schelling ([3], p. 123) calls this type of threat a ‘warning’, to distinguish it from the type of strategic threats that require commitment to be credible. Threats to exercise outside options such as eviction, departure and attack are inherently credible because they are triggered when this is in the threatener's immediate interest. These are the most relevant threats for animal systems.

## 5. Conclusion

Social partners impose constraints on an individual's behaviour through actions and threats. Actions are easy to observe; threats are much more difficult. Threats will be most prevalent in dyadic interactions, where there are large asymmetries in bargaining power or the value of group membership, and where there are effective signalling systems. Threats are implicated in stable dominance hierarchies, and where animals appear to have plenty to fight over, but overt conflict is absent. In each case, the best way to test for the presence of hidden threats is to disturb the status quo by experimentally inducing or simulating transgression, adding or removing outside options, or manipulating the information each party has about the value of group membership or outside options. Pushing cooperative interactions out of equilibrium and observing the response is an incisive technique to study cooperation because it can yield information on the evolutionary causes of inequity and the means by which animals negotiate, and reveal the full range of forces, hidden and apparent, that bind cooperative interactions together.
